# A Very Rare Complication of Acute Appendicitis: Appendicovesical Fistula

**DOI:** 10.1155/2016/4517029

**Published:** 2016-04-28

**Authors:** Deniz Alis, Cesur Samanci, Yesim Namdar, Fethi Emre Ustabasioglu, Elif Yamac, Onur Tutar, Burak Ucpinar, Bulent Onal

**Affiliations:** ^1^Istanbul University Cerrahpasa Medical Faculty Department of Radiology, 34098 Istanbul, Turkey; ^2^Haseki Research and Training Hospital, Department of Urology, 34096 Istanbul, Turkey; ^3^Istanbul University Cerrahpasa Medical Faculty Department of Urology, 34098 Istanbul, Turkey

## Abstract

Appendicovesical fistula (AVF) is an uncommon type of enterovesical fistula and a very rare complication of acute appendicitis. Herein, we report a case of 39-year-old male patient who presented with persistent urinary tract infection, recurrent abdominal pain, and pneumaturia. Imaging techniques including ultrasonography (USG), computed tomography (CT), and magnetic resonance imaging (MRI) were performed to identify the abnormality. However, definitive diagnosis of AVF was made by cystoscopy.

## 1. Introduction

AVF is a rare condition, which usually occurs following appendicitis [1, 2]. In addition to appendicitis, Crohn's disease, radiation enteritis, papillovillous adenoma of appendix, appendicular diverticulitis, cystadenocarcinoma of the appendix, and carcinoid tumors may occasionally lead to AVF [3–5]. Due to inability of common imaging modalities to detect this unique pathology and varying presenting symptoms, it is very challenging to make an early diagnosis. Imaging techniques such as ultrasound (USG), computed tomography (CT), and magnetic resonance imaging (MRI) can be used to identify this disease. Although USG is the first line diagnostic modality, CT is more sensitive and specific for detecting the AVF [6]. Treatment generally involves appendectomy and repair of the urinary bladder wall which is affected by the fistula.

## 2. Case

**Figure 1:**
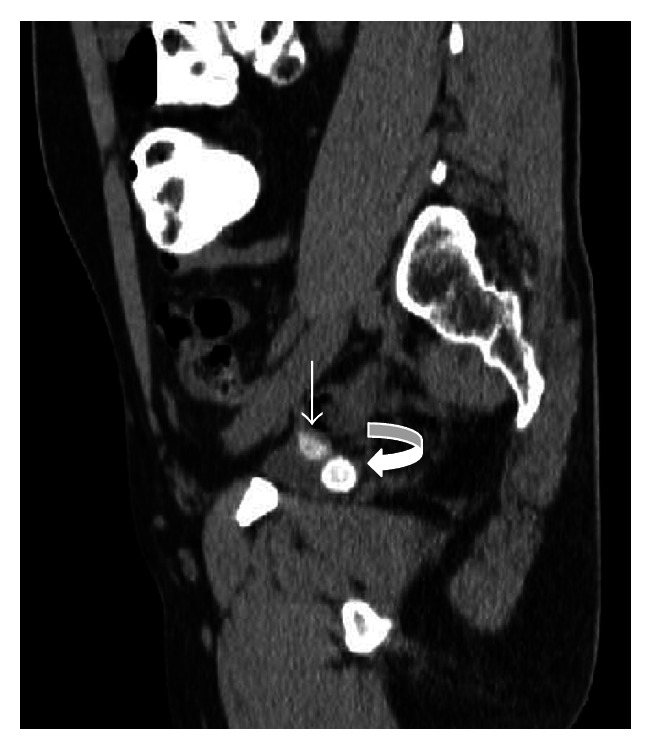
In CT images of the patient, bladder is not full enough but 2 cm hyperdense calculi are seen in posterior inferior part of bladder (curved arrow) and hyperdense fecalith image is seen in right superior posterolateral part of bladder (arrow).

**Figure 2:**
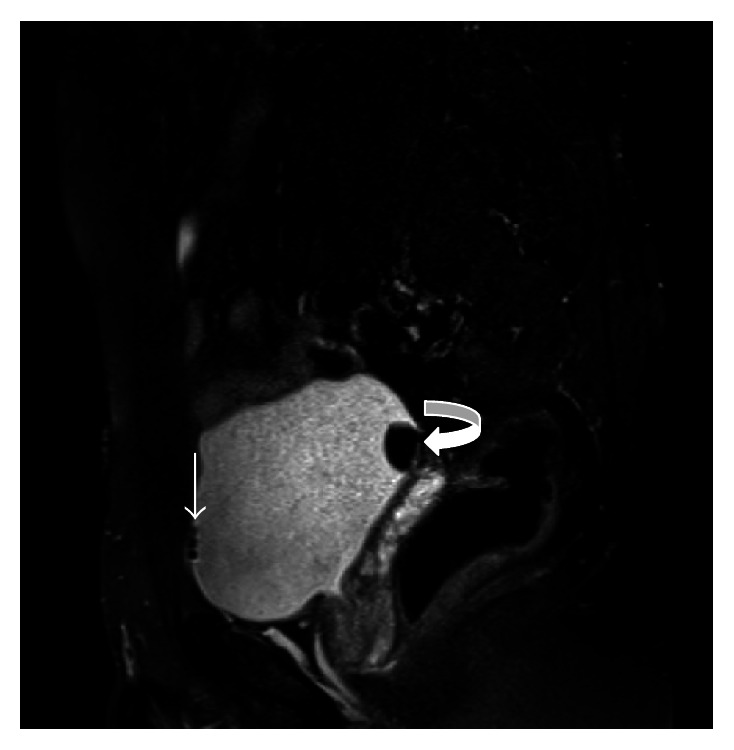
In MRI examination, there are hypointense air bubbles in anterosuperior part of bladder (arrow) and 2 cm diameter of calculi in posterosuperior part of bladder at T2-weighted image (curved arrow).

A 39-year-old man was admitted to urology department of our hospital, who had been suffering from recurrent periumbilical pain and persistent urinary tract infection. He also complained about foul smell of his urine. He had a history of acute right abdominal pain for a couple of days nearly 10 years ago but he did not receive any medical or surgical treatment. On physical examination, mild abdominal tenderness at lower quadrants was noted. Routine blood counts and biochemical analyses indicated the presence of an inflammatory condition (WBC: 13200/mm^3^ and CRP: 35). Urine analysis showed fecal content and increased number of bacteria accompanied with elevated WBC count in the urine sample, which suggested a possible enterovesical fistula. USG examination revealed mildly increased bladder wall thickness and accumulating sediments at the base of bladder. Appendix could not be visualized, but intraperitoneal fat planes around caecum showed increased echogenicity which suggested an inflammatory process. Ultrasound also revealed heterogeneous fluid collection which located lateral to the anterior aspect of the urinary bladder. Prostate and distal portions of the ureters were normal. We obtained an oral contrast CT scan to elucidate the underlying reason. CT examination demonstrated collection of fluid extending from the right anterolateral border of the bladder to the caecum. Outer diameter of the appendix exceeded normal limits (measured approximately 9 mm) and appendix was attached to the right anterolateral part of the bladder. Additionally, hyperdense fecalith image was seen in the distal portion of appendix; also there were 2 cm calculi in bladder (Figure 1). But our CT scan was not optimal for detection of possible fistula because bladder was not fully filled during CT scan. Although CT scan is the gold standard method for diagnosis of AVF, to avoid excessive radiation exposure to our patient, MRI was performed with full bladder to see fistula tract and confirm our prediagnosis. In MRI, appendix was attached to the right anterolateral part of the bladder, and a 2 cm calculus was detected in the posteroinferior part of the bladder. MRI also showed T2-weighted hypointense air bubbles which was identified in anterosuperior part of the bladder due to gravity and these findings in MRI highly suggested a possible AVF (Figure 2). To confirm the prediagnosis of AVF, the patient was referred to the urology department for cystoscopy. Cystoscopy revealed 2 cm diameter of calculi in bladder and also slit opening of fistula tract at the right lateral wall of bladder.

**Figure 3:**
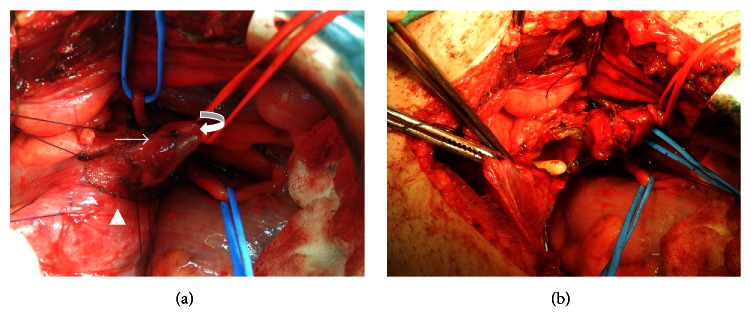
Preoperative and postoperative images of the patient (a and b). AVF (arrow) was seen in appendix (curved arrow) and was attached to bladder wall (arrow head) in preoperative images.

Appendectomy, surgical repair of bladder and catheter drainage of the area were the preferred definitive treatment for the patient (Figure 3).

## 3. Discussion

AVF is a very rare type of enterovesical fistula. Although it may accompany various conditions like Crohn's disease and malignancies, it is usually seen as a complication of acute appendicitis [2, 4, 5]. It is more commonly seen in adult males. The anatomical location of the uterus between bowel and bladder in females is thought to be main reason of this ratio [5]. Most common symptoms of AVF are recurrent urinary tract infections and pneumaturia [5]. Main challenge of AVF is late diagnosis because symptoms are usually very ambiguous. A lot of diagnostic tools have been widely used for diagnosis of AVF. USG, MRI, and CT could be used for diagnosis of AVF. Recent studies showed that CT is the most accurate diagnostic tool. Thickened bladder wall and fecalith images near the bladder are the main findings of AVF in CT scan. Also CT scan might show the fistula tract between bladder and bowel in considerable amount of cases [6]. Cystoscopy is able to diagnose AVF in 40% of the cases [7]. In our case CT, MRI, and USG were used to show possible presence of fistula, but all these methods have failed to show whether bowel and bladder were just adherent to each other due to inflammation or there was a fistula between them. Although CT and MRI were not able to show fistula tract, both of them showed secondary findings that guided us to possible AVF and in light of these findings cystoscopy has revealed fistula which opens into right lateral wall of bladder. Appendectomy, catheter drainage of the area, and repair of the bladder wall are the preferred choice for the treatment of AVF [1].

In conclusion, if a patient presents with persistent urinary infections, recurrent abdominal pain, and pneumaturia, AVF should be kept in mind for diagnosis.

Although CT urogram is generally the best diagnostic modality, cystoscopy remains an alternative method for the diagnosis of AVF in cases where the CT findings are equivocal.
